# More Competition in Mind, Better Sleep at Night? The Mediating Role of Anxiety between Competitive Attitude and Sleep Quality

**DOI:** 10.3390/ijerph20043495

**Published:** 2023-02-16

**Authors:** Kaitong Ou, Ning Ma

**Affiliations:** 1 Philosophy and Social Science Laboratory of Reading and Development in Children and Adolescents (South China Normal University), Ministry of Education, Guangzhou 510631, China; 2 Center for Sleep Research, School of Psychology, South China Normal University, Guangzhou 510631, China

**Keywords:** hypercompetitive attitude, sleep, anxiety

## Abstract

Previous studies have suggested that individuals with hypercompetitive attitude and interpersonal insecurity would have a high level of anxiety, and anxiety has been found to strongly impact on sleep quality. However, the associations between competitive attitudes and sleep quality have not been studied until now. The present study aimed to examine whether anxiety mediates the relationship between competitive attitudes and interpersonal relationships with sleep quality. This was a cross-sectional study with a total of 713 college students (age = 20.18 ± 2.16 years old; 78.8% female) recruited online to measure hypercompetitive attitude, personal development competitive attitude, interpersonal security, state anxiety and sleep quality. Path analysis models were conducted in this study. The path analysis models showed that both hypercompetitive attitude and interpersonal security had direct and indirect significant effects on poor sleep quality due to the mediating effect of state anxiety (β = 0.023, 95% bootstrapped CI: 0.005 to 0.047; β = −0.051, 95% bootstrapped CI: −0.099 to −0.010, respectively). However, personal development competitive attitude had only an indirect significant effect, but it had a negative role on poor sleep quality via state anxiety (β = −0.021, 95% bootstrapped CI: −0.042 to −0.008). The current study provided evidence that college students’ competitive attitudes would impact sleep quality and highlighted the mediating role of state anxiety. The current findings suggested that individuals shifting their hypercompetitive thinking to concentrate on ability development would benefit their mental health.

## 1. Introduction

College students in emerging adulthood (ages 18 to 25) are in a transitional and unstable period that provides the most possibility for identity exploration in academics, love, employment, and worldviews [1]. The stress, novelty, or change could temporarily cause an increased amount of comparison [2], which would somehow enhance individuals’ competitiveness [3]. In uncertain environment, the outcome of competition—winning or losing—is an important indicator contributing to self-evaluation [4]. However, in the face of competition, some individuals care more about self-discovery and self-improvement during the process than comparing themselves to others. In contrast, some individuals are obsessed with the outcome and their superiority over opponents with failure anxiety [5]. Individuals’ competitive attitude and motivation might be the factors behind these differences. Personal development competitive attitude (PDCA) and hypercompetitive attitude (HCA) are two kinds of competitive attitudes [6]. Individuals with higher PDCA focus on enjoyment and mastery of the task. They see others as helpers who provide personal discovery and learning opportunities, rather than as roadblocks to success [7]. In contrast, individuals higher in HCA have an indiscriminate desire to compete and win (and avoid losing) at any cost to maintain or enhance feelings of self-worth, with attendant orientations of manipulation, aggressiveness, exploitation, and derogation of others across a myriad of situations [8]. Individuals with HCA exhibit lower levels of intimacy satisfaction, lower self-esteem, and higher levels of neuroticism, which means that they are more likely to have mental health problems [5].

Ryckman et al. reported that hypercompetitiveness was linked to various pathological behavior [8,9]. Researchers have found that a high level of perceived class competitiveness in college students was linked to an increased risk of anxiety and depression. Perceptions of academic competitiveness raised the probability of anxiety by 70% and depression by 40% [10]. The rank-focused competitive beliefs were related to anxiety and stress. Fears of missing out, being missed and being rejected by others were strong among people who believed they must struggle for their social place and avoid inferiority [11]. Individuals with an outcome-focused competitive attitude seem more likely to have higher anxiety.

Hypercompetitiveness and insecure relationships are somehow intertwined [12]. Stapel and Koomen demonstrated that competition promoted a differentiation thinking in which differences [13] between self and others were emphasized was likely to cause social comparison. Individuals with higher HCA have a strong desire to win, which is more likely to generate upward contrast, which would bring a more detrimental impact on interpersonal interaction with less pro-social behavior, more schadenfreude, less trust and less cooperation at work [14]. Moreover, researchers suggested that people who perceive others as potential enemies would feel insecure and unsafe in their social relationships, prompting them to compete to avoid inferiority, and the feeling of insecure social relationships stimulates a variety of potential defenses, such as anxiety and low mood [15,16,17,18]. College students in emerging adulthood are gradually independent of parents, spending more time with peers and looking for high-quality interpersonal relationships [19]. Emerging adulthood is also a period for individuals to pursue job development or academic achievement involving varying degrees of competition, which may negatively impact their interpersonal relationships [20]. The negative interpersonal relationship was related to a high level of anxiety [21]. Researchers have also found that university students with greater loneliness in social interaction had a higher incidence rate of mental health problems, such as general anxiety and stress [22]. Therefore, it can be inferred that young adults with hypercompetitiveness and insecure interpersonal relationships might be more likely to experience mental disorders and problems, such as anxiety.

Individuals with anxiety often experience poor sleep quality [23]. Compared with healthy controls, individuals with a generalized anxiety disorder reported worse sleep quality, reflected in maintenance insomnia, early morning awakening, and initial insomnia [24]. Horváth et al. provided further evidence of an association between anxiety and sleep quality, finding that state anxiety was linked to an increase in sleep onset latency, which would significantly affect individuals’ subjective evaluation of sleep quality [25]. Specifically, research based on a sample of emerging adults has shown that a lower level of anxiety mediated the relationship between trait mindfulness and better sleep quality [26]. Emerging adults with a high level of anxiety are more likely to suffer from poor sleep quality, which negatively impacts their health, such as declines in immunological [27], cognitive [28] and emotional functions [29].

Sleep is an important predictor of life quality. However, individuals’ sleep quality is associated with their level of anxiety [30]. As for college students, their stress during emerging adulthood—academic/career achievement, pressure to succeed, maintaining social relationships—often results in poor sleep quality [31]. In addition, when young adults face social and life challenges, they might encounter problems of interpersonal competitiveness and communication [3], which would increase their anxiety level [10,32]. Therefore, anxiety appears to be a potential mediator in the effect of competitiveness and interpersonal relationship on the sleep quality of young adults.

In the current study, we applied a cross-sectional design to investigate the associations among competitiveness, interpersonal relationship, anxiety and sleep quality. In addition, we also explored the potential mediation role of anxiety in the influence of competitiveness and interpersonal relationship on sleep quality. Based on previous evidence, individuals who are overly focused on competition frequently experience interpersonal issues [14,20], which would increase the possibility of anxiety [10,11,22], a risk factor for poor sleep [25,26], we hypothesized that both competitive attitude and interpersonal insecurity would have a significant effect on sleep quality, and state anxiety would act as a mediator between the relationships.

## 2. Methods

### 2.1. Participants and Procedure

Data were collected between October 2019 and November 2019. We used a convenience sampling procedure to recruit participants on the Internet. The survey data were collected anonymously, and the participants voluntarily decided to take part in this research. The study’s inclusion criteria were as follows: age 18 to 24; college student. Exclusion criteria were as follows: having clinical mental disorders, clinical sleep disorders and medical treatments during the study or one month before participating in the study (mentioned in the instruction of the survey). Based on the criteria, 768 valid college students were chosen for the survey, and 55 were excluded because of incomplete data. As a result, 713 participants were analyzed as the final sample (78.8% female, 20.18 ± 2.16 years old).

After providing informed consent, participants were asked to answer a collection of online questionnaires in the fixed order, including the Competitive Attitude Scale–Chinese version, Security Questionnaire, State–Trait Anxiety Inventory, and Sleep Quality Scale. The time participants completed the questionnaires ranged from 10 to 15 min. The study was approved by the Ethics Committee of South China Normal University and was conducted in accordance with the Declaration of Helsinki.

### 2.2. Measures

#### 2.2.1. Competitive Attitudes

Competitive attitudes were assessed using a 27-item Chinese version of Competitive Attitude Scale (CAS-C), developed by Chen and Liu in 2003 [33] from the Hypercompetitive Attitude Scale and the Personal Development Competitive Attitude Scale of Ryckman et al. [7,8]. Some items had been modified to better fit Chinese culture. The CAS-C consists of a 14-item personal development competitive attitude subscale (e.g., “I enjoy competition because it gives me a chance to discover my abilities”), and a 13-item hypercompetitive attitude subscale (e.g., “It’s a dog-eat-dog world. If you don’t get the better of others, they will surely get the better of you”). Four reversed items are randomly distributed over the scale (e.g., “I do not see my opponents in competition as my enemies”). Each item is rated on a 5-point Likert scale from 1 (strongly disagree) to 5 (strongly agree). A higher score indicates a higher competitive attitude. The personal development competitive attitude scale score ranges from 14 to 70, and the hypercompetitive attitude subscale score ranges from 13 to 65. In this study, Cronbach’s alpha for the personal development competitive attitude subscale was 0.88, and for the hypercompetitive attitude subscale, it was 0.74.

#### 2.2.2. Interpersonal Security

To measure interpersonal security, we employed the interpersonal security subscale of the Security Questionnaire created by Cong and An in 2004 [34]. The Security Questionnaire is a self-rating scale with two subscales: interpersonal security and certainty of control. The scale has a total of 16 items, with 8 items for each of the 2 subscales. Interpersonal security reflects the security experiences of individuals during interpersonal communication (e.g., “I am afraid of establishing and maintaining close relationships with others”). Certainty of control measures an individual’s life prediction, sense of certainty, and sense of control (e.g., “I feel that life is full of uncertainty and unpredictability”). Each item is rated on 5 points, from 1 (strongly agree) to 5 (strongly disagree). The range of scores for each subscale is 8 to 40, with higher scores indicating high security. The Cronbach’s alpha for interpersonal security was 0.82, and for the certainty of control was 0.85 in this study.

#### 2.2.3. State Anxiety

The State–Trait Anxiety Inventory (Form Y) was developed by Spielberger in 1983 [35] and translated into Chinese by Shen in 1988 [36]. In this study, the state anxiety subscale of the Chinese Version of State–Trait Anxiety Inventory was used to assess state anxiety. The State–Trait Anxiety Inventory is a 40-item inventory including 2 separate subscales to assess state and trait anxiety, respectively. The state anxiety items measure how individuals feel right now (e.g., “I am tense”), while the trait anxiety items assess how people feel in general (e.g., “I feel nervous and restless”). The whole scale contains 20 reverse items (e.g., “I feel calm”), and the scoring must be reversed. Each item is rated on a 4-point scale from 1 (not at all) to 4 (very so much), with a maximum score of 80 for each subscale. Higher scores indicate higher anxiety. The Cronbach’s alpha for state anxiety in this study was 0.93, and for trait anxiety, it was 0.82.

#### 2.2.4. Sleep Quality

Sleep quality was evaluated using a 28-item Chinese version of the Sleep Quality Scale developed by Yi et al. in 2006 [37]. It measures adults’ sleep quality of the previous month and gives out a global score. The scale consists of 6 factors, including daytime dysfunction (e.g., “Difficulty in thinking due to poor sleep”), restoration after sleep (e.g., “Relief of fatigue after sleep”), difficulty in falling asleep (e.g., “Difficulty in getting back to sleep after nocturnal awakening”), difficulty in getting up (e.g., “Wish for more sleep after getting up”), satisfaction with sleep (e.g., “Deep sleep”), and difficulty in maintaining sleep (e.g., “Waking up easily due to noise”). Each item is rated on a 4-point Likert scale from 0 (few) to 3 (almost always). Scores on the restoration after sleep and satisfaction with sleep items must be reversed. The total score ranges from 0 to 84. Higher scores represent lower sleep quality. In this study, Cronbach’s alpha was 0.88.

### 2.3. Analysis

Based on the research hypothesis, the subscales score of hypercompetitive attitude, personal development competitive attitude, interpersonal security, state anxiety, and total sleep quality scores were chosen as the final analyzed data. The Harman single-factor test was used to analyze the common method bias in order to avoid the common method deviation caused by the self-report questionnaire survey. The factor analysis results showed that the first common factor explained 16.83% of the variance (less than 40%), indicating that a common method bias was unlikely to influence our results.

Descriptive data analysis was conducted to reveal the basic characteristics of the variables. Bivariate Pearson analysis was conducted to calculate the correlations between hypercompetitive attitude, personal development competitive attitude, interpersonal security, state anxiety and sleep quality variables.

**Figure 1:**
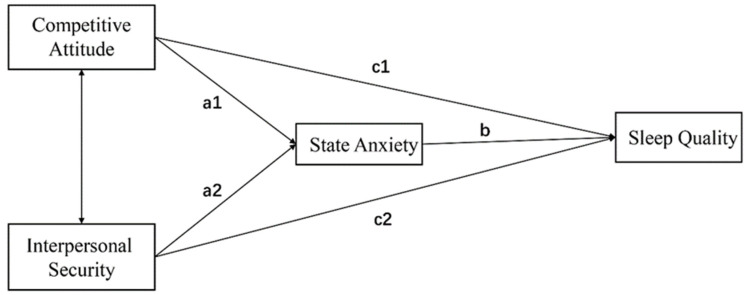
Path analysis model conducted in the current study. Notes: Double-headed arrows represent the association between variables, and single-headed arrows represent prediction routes.

Mplus v8.0 was used to conduct path analysis models. For our study, the path analysis model involved two independent variables, one mediator and one outcome variable. We conducted two models which consider competitive attitude (i.e., hypercompetitive attitude in the first model while personal development competitive attitude in the second model) and interpersonal security as independent variables, state anxiety as a mediator and sleep quality as an outcome variable in both models. As shown in Figure 1, both models examined the direct paths from one of the competitive attitudes and interpersonal security to sleep quality, as well as the indirect paths from one of the competitive attitudes and interpersonal security to sleep quality through state anxiety. Gender and college year were statistically controlled in the path analysis.

## 3. Results

### 3.1. Descriptive Analysis and Bivariate Correlation

The final study sample included 713 participants, among which 562 (78.8%) were women, and 151 (21.2%) were men. Descriptive statistics for all measures and the results of the bivariate correlational analysis are presented in Table 1. Except for the correlation between personal development competitive attitude and sleep quality, the scores of all measures were significantly correlated with each other. Specifically, interpersonal security was negatively correlated with state anxiety (*r* = −0.41, *p* < 0.01), sleep quality (*r* = −0.18, *p* < 0.01) and hypercompetitive attitude (*r* = −0.27, *p* < 0.01), and positively correlated with personal development competitive attitude (*r* = 0.32, *p* < 0.01). State anxiety was positively correlated with sleep quality (*r* = 0.18, *p* < 0.01) and hypercompetitive attitude (*r* = 0.30, *p* < 0.01), and negatively correlated with personal development competitive attitude (*r* = −0.27, *p* < 0.01). Hypercompetitive attitude was positively correlated with sleep quality (*r* = 0.16, *p* < 0.01), and negatively correlated with personal development competitive attitude (*r* = −0.16, *p* < 0.01). However, personal development competitive attitude had an insignificantly negative correlation with sleep quality (*r* = −0.02, *p* = 0.65).

### 3.2. Path Analysis

**Figure 2:**
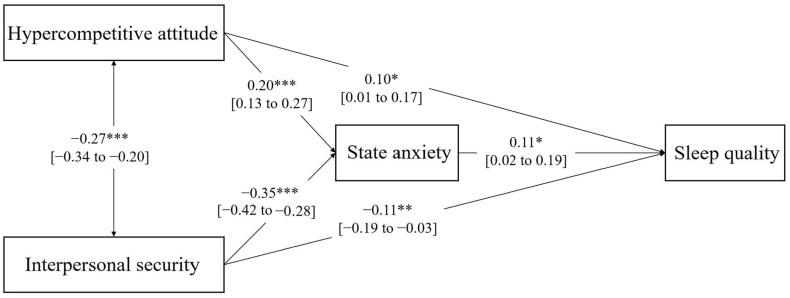
Path analysis model for hypercompetitive attitude with standardized coefficients. Note: The solid line refers to the statistically significant association. * *p* < 0.05, ** *p* < 0.01, *** *p* < 0.001. 95% CI are detailed between brackets.

Figure 2 showed the first path analysis model for hypercompetitive attitude with path coefficients. Specifically, the direct effect of hypercompetitive attitude on sleep quality was positive and significant (*β* = 0.10, *p* < 0.05). Additionally, the direct effect of interpersonal security on sleep quality was significantly negative (*β* = −0.11, *p* < 0.01). The direct effect of hypercompetitive attitude on state anxiety was significantly positive (*β* = 0.20, *p* < 0.001), and interpersonal security on state anxiety were significantly negative (*β* = −0.35, *p* < 0.001). The direct effect of state anxiety on sleep quality was significantly positive (*β* = 0.11, *p* < 0.05). The indirect effects of hypercompetitive attitude and interpersonal security on sleep quality through state anxiety were significant (a1*b = 0.023, 95% bootstrapped CI: 0.005 to 0.047; a2*b = −0.051, 95% bootstrapped CI: −0.099 to −0.010).

**Figure 3:**
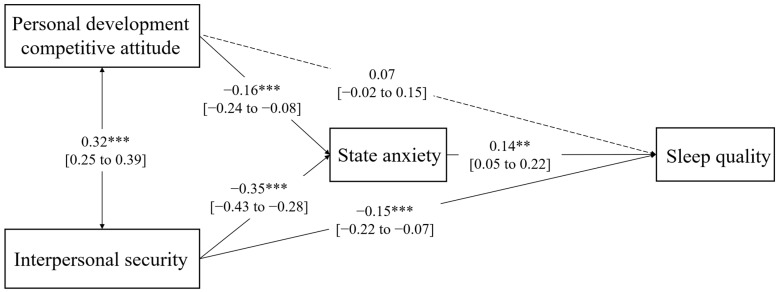
Path analysis model for personal development competitive attitude with standardized coefficients. Note: The solid line refers to the statistically significant path. The dashed line refers to the statistically insignificant path. ** *p* < 0.01, *** *p* < 0.001. 95% CI are detailed between brackets.

The second path analysis model with personal development competitive attitude is presented in Figure 3. Specifically, the direct effect of interpersonal security on sleep quality was significantly negative (*β* = −0.15, *p* < 0.001). The direct effect of personal development competitive attitude and interpersonal security on state anxiety was significantly negative (*β* = −0.16, *p* < 0.001; *β* = −0.35, *p* < 0.001, respectively). The direct effect of state anxiety on sleep quality was significantly positive (*β* = 0.14, *p* < 0.01). The indirect effects of personal development competitive attitude and interpersonal security on sleep quality through state anxiety were significant (a1*b = −0.021, 95% bootstrapped CI: −0.042 to −0.008; a2*b = −0.066, 95% bootstrapped CI: −0.116 to −0.025).

In summary, mediation effects of state anxiety were discovered in the first path analysis model. The findings demonstrated that a hypercompetitive attitude and interpersonal security were both directly and indirectly related to sleep quality via the state anxiety pathway. However, in the second path analysis model, state anxiety acted as a mediator between interpersonal security and sleep quality while working as a suppression variable between personal development competitive attitude and sleep quality [38,39]. As in the first model, interpersonal security exhibited a direct and indirect relationship with sleep quality via state anxiety. Nevertheless, personal development competitive attitude demonstrated only an indirect relationship with sleep quality via state anxiety.

## 4. Discussion

Individuals with hypercompetitive attitudes have been found to have lower levels of optimal psychological health [9]. However, the associations between competitive attitudes and sleep quality have not been investigated. The current study aimed to investigate the role of state anxiety in mediating the relationships between competitive attitudes and interpersonal security with sleep quality among college students. As predicted, our findings revealed that individuals with hypercompetitive attitude and interpersonal insecurity are more likely to experience poor sleep quality due to the increased state anxiety, while individuals with personal development competitive attitude would experience less state anxiety and be less likely to have poor sleep quality. To our knowledge, this is the first study that assessed the relationship between competitive attitude and sleep quality, and the current findings suggest the importance of the way to view competition on college students’ mental health.

Our study found that a higher hypercompetitive attitude would increase college students’ state anxiety, which leads to poor sleep quality. Previous studies have noted hypercompetitive individuals’ intense pursuit of success would result in time and energy over-expenditure and lead to anxiety experience [9]. Moreover, individuals who wish to demonstrate their competence are afraid of failure [40] and related to high anxiety and worry [41]. Most importantly, worrying about performance or failure in competition may trigger autonomic arousal and emotional distress and cause increased anxiety. The anxious status would cause individuals to prioritize the allocation of attentional resources to worries and threats in mind after going to bed, resulting in poor sleep quality [42]. However, a personal development competitive attitude would decrease individuals’ state anxiety. A previous study noted that individuals who focus on the mastery of tasks and ability improvement have low levels of anxiety (e.g., worry and emotionality) [41], which reduces the likelihood of poor sleep quality [42].

The current findings also indicated a significant indirect association between interpersonal security and poor sleep quality via state anxiety. According to the intrinsic processes interpersonal emotion regulation model [43], social contact is a kind of emotion regulation process that helps to reduce individuals’ anxiety [44]. In addition, a positive interpersonal relationship provides social support for individuals, which helps to reduce their anxiety [44]. However, peer rejection and negative peer experiences increase individuals’ anxiety [45]. Furthermore, individuals with a high level of anxiety would be easily overwhelmed by physiological hyperarousal, intrusive thoughts, and excessive, uncontrollable worry when attempting to fall asleep, resulting in poor sleep [46]. In other words, individuals with low interpersonal security are more likely to experience increased anxiety, impairing their sleep quality.

We also found that both hypercompetitive attitude and interpersonal relationships have significant direct effects on poor sleep quality, which was partially outside of our expectations. The results indicated that other psychological mechanisms might contribute to the impacts of hypercompetitive attitude and interpersonal security on sleep quality. Concerning the direct effect of hypercompetitive attitude on sleep quality, previous research has indicated that individuals with hypercompetitive attitudes easily generate high levels of stress, hostility, rumination and distress [9,47,48], which had negative impacts on sleep quality [42,49,50,51,52]. Besides the mediating effect of anxiety between interpersonal security and sleep quality, previous studies have also noted that depression could mediate the association between aversive social relationships and poor sleep quality [53]. Moreover, the perception of inadequate social interaction would lead to loneliness, which had a robust association with poorer sleep quality [54].

As for practical implications, it might be helpful to include competitive attitudes and interpersonal security in improving college students’ sleep quality. Anxiety is a well-known risk factor for poor sleep quality [23]. According to the cognitive model developed by Beck, individuals’ irrational perceptions of events may set off excessive reactions and even mental disorders such as anxiety [55]. Therefore, college students’ cognition and attitude toward competition are especially crucial when encountering stress in emerging adulthood. Educators can encourage college students to emphasize their personal growth, rather than competition outcomes, and to view their peers as a source of support rather than social comparison [10,56], which helps to keep anxiety within a reasonable range. It will contribute to improving college students’ mental health, such as sleep quality.

The study has some limitations. First, the cross-sectional design of the study precludes us from making causal inferences in the relationships between hypercompetitive attitude, interpersonal security, state anxiety and sleep quality. Moreover, a bidirectional relationship might exist in which poor sleep quality exacerbates anxiety [57], thus aggravating poor sleep quality. Further studies can employ longitudinal or randomized control designs to further evaluate sleep quality with hypercompetitiveness, interpersonal relationships and anxiety over a longer time.

Second, the probable psychological mechanisms underlying the direct effects of hypercompetitive attitude and interpersonal security on sleep quality remain unproven. Although we have made some conjectures regarding possible underlying mechanisms, further investigation of the mechanism between these variables is needed to increase our understanding of the relationship between hypercompetitiveness, interpersonal relationships and sleep quality among college students.

Finally, in order to keep participants’ attention focused and minimize the time it took to complete the questionnaire, we did not ask too many questions to collect their socio-demographic information except age, gender and college year. Other socio-demographic information such as students’ location, family structure, and family socioeconomic status of the participants was unknown. Researchers found that teenagers from two-parent families and those whose family has a higher income-to-needs ratio sleep better [58,59]. The family structure and family socioeconomic status may influence the relationships between our variables. Future researchers could investigate the role of the socio-demographic information in the relationships between competitive attitude, interpersonal relationships, state anxiety and sleep quality.

## 5. Conclusions

The current study applied a path analysis model to examine the role of state anxiety in mediating the relationships between hypercompetitive attitude and interpersonal security with sleep quality among college students. The findings indicated that both hypercompetitive attitude and interpersonal insecurity had significant direct and indirect effects on poor sleep quality through state anxiety. Altering hypercompetitive thinking to focus on personal development in competition, improving interpersonal security in social relationships and using mindful techniques to lower anxiety levels would benefit individuals’ sleep quality. Future studies should investigate the direction and other potential psychological mechanisms of the relationship between hypercompetitiveness and interpersonal security with sleep quality.

## Figures and Tables

**Table 1:** Descriptive statistics and bivariate correlation coefficients between measured variables (n = 713).

	Mean ± SD	1	2	3	4	5
1. Interpersonal security	26.72 ± 5.54	-				
2. State anxiety	39.75 ± 10.44	−0.41 **	-			
3. Sleep quality	58.15 ± 7.63	−0.18 **	0.18 **	-		
4. Hypercompetitive attitude	32.27 ± 7.14	−0.27 **	0.30 **	0.16 **	-	
5. Personal development competitive attitude	50.21 ± 7.74	0.32 **	−0.27 **	−0.02	−0.16 **	-

Note: ** *p* < 0.01.

## Data Availability

The datasets of the current study are available from the corresponding authors on reasonable request.

## References

[1] Arnett J.J. (2000). Emerging Adulthood: A Theory of Development from the Late Teens through the Twenties. Am. Psychol..

[2] Gibbons F.X., Buunk B.P. (1999). Individual Differences in Social Comparison: Development of a Scale of Social Comparison Orientation. J. Pers. Soc. Psychol..

[3] Garcia S.M., Tor A., Schiff T.M. (2013). The Psychology of Competition: A Social Comparison Perspective. Perspect. Psychol. Sci..

[4] Meeker B.F. (1990). Cooperation, Competition, and Self-Esteem: Aspects of Winning and Losing. Hum. Relat..

[5] Ersilia M. (2018). The Competitive Attitude Scale (CAS): A Multidimensional Measure of Competitiveness in Adolescence. J. Psychol. Clin. Psychiatry.

[6] Ryckman R.M., Libby C.R., van den Borne B., Gold J.A., Lindner M.A. (1997). Values of Hypercompetitive and Personal Development Competitive Individuals. J. Pers. Assess..

[7] Ryckman R.M., Hammer M., Kaczor L.M., Gold J.A. (1996). Construction of a Personal Development Competitive Attitude Scale. J. Pers. Assess..

[8] Ryckman R.M., Hammer M., Kaczor L.M., Gold J.A. (1990). Construction of a Hypercompetitive Attitude Scale. J. Pers. Assess..

[9] Ryckman R.M., Thornton B., Butler J.C. (1994). Personality Correlates of the Hypercompetitive Attitude Scale: Validity Tests of Horney’s Theory of Neurosis. J. Pers. Assess..

[10] Posselt J.R., Lipson S.K. (2016). Competition, Anxiety, and Depression in the College Classroom: Variations by Student Identity and Field of Study. J. Coll. Stud. Dev..

[11] Gilbert P., McEwan K., Bellew R., Mills A., Gale C. (2009). The Dark Side of Competition: How Competitive Behaviour and Striving to Avoid Inferiority Are Linked to Depression, Anxiety, Stress and Self-Harm. Psychol. Psychother. Theory Res. Pract..

[12] Thornton B., Ryckman R.M., Gold J.A. (2011). Hypercompetitiveness and Relationships: Further Implications for Romantic, Family, and Peer Relationships. Psychology.

[13] Stapel D.A., Koomen W. (2005). Competition, Cooperation, and the Effects of Others on Me. J. Pers. Soc. Psychol..

[14] Ding M., Liu Y., Qing H. (2018). The Interpersonal Impact of Social Comparison. Psychology.

[15] Dykman B.M. (1998). Integrating Cognitive and Motivational Factors in Depression: Initial Tests of a Goal-Orientation Approach. J. Pers. Soc. Psychol..

[16] Gilbert P. (1992). Human Nature and Suffering.

[17] Gilbert P. (2005). Compassion and Cruelty: A Biopsychosocial Approach. Compassion: Conceptualisations, Research and Use in Psychotherapy.

[18] Gilbert P. (2005). Social Mentalities: A Biopsychosocial and Evolutionary Approach to Social Relationships. Interpersonal Cognition.

[19] Halfon N., Forrest C.B., Lerner R.M., Faustman E.M. (2018). Handbook of Life Course Health Development.

[20] Hibbard D.R., Buhrmester D. (2010). Competitiveness, Gender, and Adjustment Among Adolescents. Sex Roles.

[21] Vaughn A.A., Drake R.R., Haydock S. (2016). College Student Mental Health and Quality of Workplace Relationships. J. Am. Coll. Health.

[22] Richardson T., Elliott P., Roberts R. (2017). Relationship between Loneliness and Mental Health in Students. J. Public Ment. Health.

[23] Chellappa S.L., Aeschbach D. (2022). Sleep and Anxiety: From Mechanisms to Interventions. Sleep Med. Rev..

[24] Brenes G.A., Miller M.E., Stanley M.A., Williamson J.D., Knudson M., McCall W.V. (2009). Insomnia in Older Adults with Generalized Anxiety Disorder. Am. J. Geriatr. Psychiatry.

[25] Horváth A., Montana X., Lanquart J.-P., Hubain P., Szűcs A., Linkowski P., Loas G. (2016). Effects of State and Trait Anxiety on Sleep Structure: A Polysomnographic Study in 1083 Subjects. Psychiatry Res..

[26] Bogusch L.M., Fekete E.M., Skinta M.D. (2016). Anxiety and Depressive Symptoms as Mediators of Trait Mindfulness and Sleep Quality in Emerging Adults. Mindfulness.

[27] Besedovsky L., Lange T., Born J. (2012). Sleep and Immune Function. Pflüg. Arch.—Eur. J. Physiol..

[28] Diekelmann S. (2014). Sleep for Cognitive Enhancement. Front. Syst. Neurosci..

[29] Goldstein A.N., Walker M.P. (2014). The Role of Sleep in Emotional Brain Function. Annu. Rev. Clin. Psychol..

[30] Nechita D., Nechita F., Motorga R. (2018). A Review of the Influence the Anxiety Exerts on Human Life. Rom. J. Morphol. Embryol. Rev. Roum. Morphol. Embryol..

[31] Oliver M.D., Baldwin D.R., Maples O.M., Hakeem F.E., Datta S. (2018). Sleep Quality and Duration Best Predict Quality of Life in College Students. Sleep Vigil..

[32] Li J., Li J., Jia R., Wang Y., Qian S., Xu Y. (2020). Mental Health Problems and Associated School Interpersonal Relationships among Adolescents in China: A Cross-Sectional Study. Child Adolesc. Psychiatry Ment. Health.

[33] Chen G.P., Lu F. (2003). The Competitive Attitude Scale. J. Psychol. Sci..

[34] Cong Z., An L.J. (2004). Developing of Security Questionnaire and Its Reliability and Validity. Chin. J. Ment. Health.

[35] Spielberger C.D. (1983). Manual for the State-Trait Anxiety Inventory (STAI) (Form Y).

[36] Shen Y. (1988). Psychiatry.

[37] Yi H., Shin K., Shin C. (2006). Development of the Sleep Quality Scale. J. Sleep Res..

[38] MacKinnon D.P., Krull J.L., Lockwood C.M. (2000). Equivalence of the Mediation, Confounding and Suppression Effect. Prev. Sci. Off. J. Soc. Prev. Res..

[39] Wen Z., Ye B. (2014). Analyses of Mediating Effects: The Development of Methods and Models. Adv. Psychol. Sci..

[40] Edwards O.V. (2014). Differentiating Performance Approach Goals and Their Unique Effects. Univers. J. Educ. Res..

[41] Stan A., Oprea C. (2015). Test Anxiety and Achievement Goal Orientations of Students at a Romanian University. Procedia—Soc. Behav. Sci..

[42] Harvey A.G. (2002). A Cognitive Model of Insomnia. Behav. Res. Ther..

[43] Zaki J., Williams W.C. (2013). Interpersonal Emotion Regulation. Emotion.

[44] Hofmann S.G. (2014). Interpersonal Emotion Regulation Model of Mood and Anxiety Disorders. Cogn. Ther. Res..

[45] La Greca A.M., Harrison H.M. (2005). Adolescent Peer Relations, Friendships, and Romantic Relationships: Do They Predict Social Anxiety and Depression?. J. Clin. Child Adolesc. Psychol..

[46] Kirwan M., Pickett S.M., Jarrett N.L. (2017). Emotion Regulation as a Moderator between Anxiety Symptoms and Insomnia Symptom Severity. Psychiatry Res..

[47] Chan J.Y., Gerstein L.H., Kinsey R., Fung A.L. (2018). Asian Adults’ Hypercompetitiveness and Distress: The Mediating Role of a Negative Problem-Solving Orientation. Curr. Psychol..

[48] Grant H., Dweck C.S. (2003). Clarifying Achievement Goals and Their Impact. J. Pers. Soc. Psychol..

[49] Lund H.G., Reider B.D., Whiting A.B., Prichard J.R. (2010). Sleep Patterns and Predictors of Disturbed Sleep in a Large Population of College Students. J. Adolesc. Health.

[50] Rezaei M., Khormali M., Akbarpour S., Sadeghniiat-Hagighi K., Shamsipour M. (2018). Sleep Quality and Its Association with Psychological Distress and Sleep Hygiene: A Cross-Sectional Study among Pre-Clinical Medical Students. Sleep Sci..

[51] Tsuchiyama K., Terao T., Wang Y., Hoaki N., Goto S. (2013). Relationship between Hostility and Subjective Sleep Quality. Psychiatry Res..

[52] Qiu W.-F., Ma J.-P., Xie Z.-Y., Xie X.-T., Wang C.-X., Ye Y.-D. (2022). Online Risky Behavior and Sleep Quality among Chinese College Students: The Chain Mediating Role of Rumination and Anxiety. Curr. Psychol..

[53] Kent R.G., Uchino B.N., Cribbet M.R., Bowen K., Smith T.W. (2015). Social Relationships and Sleep Quality. Ann. Behav. Med..

[54] Matthews T., Danese A., Gregory A.M., Caspi A., Moffitt T.E., Arseneault L. (2017). Sleeping with One Eye Open: Loneliness and Sleep Quality in Young Adults. Psychol. Med..

[55] Beck A.T. (1976). Cognitive Therapy and the Emotional Disorders.

[56] Twenge J.M., Gentile B., DeWall C.N., Ma D., Lacefield K., Schurtz D.R. (2010). Birth Cohort Increases in Psychopathology among Young Americans, 1938–2007: A Cross-Temporal Meta-Analysis of the MMPI. Clin. Psychol. Rev..

[57] Zou P., Wang X., Sun L., Liu K., Hou G., Yang W., Liu C., Yang H., Zhou N., Zhang G. (2020). Poorer Sleep Quality Correlated with Mental Health Problems in College Students: A Longitudinal Observational Study among 686 Males. J. Psychosom. Res..

[58] Troxel W.M., Lee L., Hall M., Matthews K.A. (2014). Single-Parent Family Structure and Sleep Problems in Black and White Adolescents. Sleep Med..

[59] Rocha S., Almeida D.M., Chiang J.J., Cole S.W., Irwin M.R., Seeman T., Fuligni A.J. (2022). The Relationship Between Family Socioeconomic Status and Adolescent Sleep and Diurnal Cortisol. Psychosom. Med..

